# Nanomechanics and Nanorheology of Microgels at Interfaces

**DOI:** 10.3390/polym10090978

**Published:** 2018-09-03

**Authors:** Sebastian Backes, Regine von Klitzing

**Affiliations:** 1 Stranski-Laboratorium für Physikalische und Theoretische Chemie, Technische Universität Berlin, Strasse des 17. Juni 124, D–10623 Berlin, Germany; sebastian.backes@tu-berlin.de; 2 Soft Matter at Interfaces, Department of Physics, TU Darmstadt, Alarich-Weiss-Strasse 10, D–62487 Darmstadt, Germany

**Keywords:** swelling ability, nanomechanics, nanorheology, stimuli-sensitive gels, microgels, hydrogels

## Abstract

The review addresses nanomechanics and nanorheology of stimuli responsive microgels adsorbed at an interface. In order to measure the mechanical properties on a local scale, an atomic force microscope is used. The tip presents an indenter with a radius of curvature of a few 10 s of nm. Static indentation experiments and dynamic studies with an excited cantilever are presented. The effect of several internal and external parameters on the mechanical properties is reviewed. The focus is on the correlation between the swelling abilities of the gels and their mechanical properties. Several results are surprising and show that the relationship is not as simple as one might expect.

## 1. Introduction

Hydrogels are defined as cross-linked polymeric networks that are able to swell in water. In the review, mainly chemical cross-linked hydrogels will be considered, which show a volume phase transition triggered by an external stimulus, mainly temperature [1]. Depending on their composition, other external stimuli can be for instance ionic strength [2] or pH value [3]. If functional groups (e.g., chromophores) or metal particles are embedded the gel can also interact with external fields like light [4] or magnetic fields [5,6].

Besides macrogels [7], in the last 20 years microgels got more and more impact [8,9,10,11,12]. The advantage of microgels are the faster response kinetics to external stimuli due to their smaller distances [13] and the ease in preparation of coatings just by adsorption of microgels. The transition is less sharp in comparison to the one of macrogels due to a larger heterogeneity of sub chain length [14]. Microgel particles are mostly synthesised by emulsion or precipitation polymerisation. For instance, in poly(*N*–isopropyl–acrylamide) (PNIPAM) microgels NIPAM is often cross-linked by *N*,*N*′–methylene–bis–acrylamide (BIS) [9]. The microgels have a lower critical solution temperature (LCST) of about 32°, where the polymer–polymer interactions start over-compensating the hydration of the polymer. Due to the mentioned less sharp transition in the case of microgels, the LCST is often called volume phase transition temperature (VPTT). The copolymerisation with charged units like acrylic acid (AAc) (→P(NIPAM–co–AAc)) leads to an increase in VPTT, since the charge counteract a polymer collapse [8,9]. Another charged monomer, which is often used is methacrylic acid (MAA) [15]. Usually the increase in charge density of the microgels leads to an increase of microgel size [15,16].

For the preparation of stimuli-sensitive coatings, microgels are deposited at a surface. Lyon and coworkers fixed microgel particles on poly(ethylene terephthalate) (PET) surface by spin coating and covalent tethering afterwards [17]. A 2D monolayer of PNIPAM microgel particles with a regular distance was obtained by Kawaguchi and coworkers [18]. Serpe et al. prepared multilayers of negatively charged microgel particles and polycations via spin coating them alternately [19]. After adsorption, the microgels show a compression in volume by about one order of magnitude [20]. The swelling ability is reduced. For instance, adsorbed MAA containing microgels swell by about factor 10, which is less than in the respective bulk solution [15]. This reduction in swelling ability due to adsorption becomes more pronounced with increasing cross-linker density as reported for PNIPAM microgels [21]. Nevertheless, adsorbed microgels show still a reversible swelling/shrinking in dependence of temperature or pH. In the case of microgels of pure PNIPAM or of PNIPAM copolymerised with a low amount of comonomer (e.g., AAc), the VPTT does not change after adsorption. In the case of higher amounts of comonomer, the VPTT might change with respect to the bulk phase. For instance, the copolymerisation with AAc leads to *two* transitions (at about 32 °C and at about 45 °C) in the bulk phase which indicates microphase separation between the AAc rich and the AAc poor phase. After adsorption onto a solid wafer, the microgels show only *one* transition between 32 and 45 °C which indicates an interdigitation of the different microphase domains due to compression [16]. Another consequence of the compression is the slowing down of the dynamics within the microgels [22,23].

Mechanical properties of coatings like microgel layers are interesting for e.g., cell adhesion and for haptics as for tactile feed–back devices. For studying nanomechanical properties of the microgels, one option is to do static indentation and dynamic indentation measurements with an atomic force microscope (AFM). Therefore, the microgels have to be adsorbed at a solid surface. The advantage of using AFM indentation measurements in comparison to a bulk rheometer or surface rheometer is that the mechanical properties can be measured with a high spatial resolution in the range of the radius of the AFM tip. Intuitively, the swelling ability is correlated with the mechanical properties, and the review addresses this issue. A hypothesis might be that a high amount of swelling would directly decrease the stiffness of the gel. The review shows that this is valid for many gels, but there is also often a strong deviation from this behaviour.

## 2. Atomic Force Microscopy

Atomic force microscopy (AFM) has become a valuable technique to access the mechanical properties of microgels. By recording stress-strain curves on the microscale, elastic moduli can be determined. The application of an oscillatory stress allows the calculation of the dynamic (or complex) modulus, which characterises viscoelastic materials. The fact that AFM experiments can be done in liquid environment makes them especially suitable, and most mechanical microgel studies are performed on swollen microgels immersed in water or other solvents.

### 2.1. Static Force Measurements

The elastic modulus E can be determined in static force measurements. Therefore, the tip approaches the sample in contact mode (i.e., not oscillating) with a well defined velocity until a certain deflection of the cantilever is reached. Then the tip is retracted again. Before the measurement, the cantilever has to be calibrated with a force curve on a hard surface to determine the cantilever deflection (inverse optical lever sensitivity, InvOLS) and the exact spring constant. This is necessary to be able to convert a measured cantilever deflection value into the force that acts on the sample. The harder a sample is, the faster the force increases upon indentation, while soft and compliant samples exert lower forces at a given indentation.

The Hertzian theory of non-adhesive elastic contact [24] provides a relation between indentation depth δ, force *F* and elastic Modulus *E*

(1) $$F=\frac{4E\sqrt{R}}{3(1-\nu^{2})}\delta^{3/2}$$

for a spherical indenter with radius *R* and a sample with a Poisson ratio ν. For a conical indenter with a half cone angle of $90^{{}^{\circ}}-\theta$,

(2) $$F=\frac{2E}{\tan\theta \cdot \pi }\delta^{2}.$$

As there is usually a strong adhesion after the contact, only the approach curves are suitable for analysis.

The Poisson ratio is the ratio of transverse strain to axial strain. In studies on microgels it is usually assumed to be 0.5, which is the value for materials, where the increase in volume caused by stretching (axial strain) is compensated by the decrease in volume due to the transverse diminution (transverse strain) [16,21,25,26,27,28,29,30]. While this is valid for many samples, there is evidence of soft materials with lower Poisson ratios. An overestimation of the Poisson ratio would lead to an underestimation of the elastic modulus by nearly 18% (for ν=0.3) or 11% (for ν=0.4) [31].

The Hertz model assumes the indentation of an infinite half-space. Large errors by up to one order of magnitude can result when this model is applied to thin samples [32,33,34]. Therefore, only the first part of the force curve with an indentation depth in the range 10%–20% of the microgel height can be used to prevent an impact of the substrate [26,35]. Because of the small size of microgels, data points up to 10% of the microgel height might not be enough for a profound analysis. A model developed by Dimitriadis et al. [25] takes the finite sample thickness into account, and has been successfully applied to microgel samples [16,21]. For a microgel which is bonded to the substrate, *E* can be calculated by

(3) $$E=\frac{9F}{16}\times \frac{1}{R^{1/2}\delta^{3/2}\left(1+1.133\chi +1.283\chi^{2}+0.769\chi^{3}+0.0975\chi^{4}\right)}$$

where $\chi =\sqrt{R\delta }/h$.

There are several other extensions of the Hertz model that account for adhesive or repulsive interaction between tip and sample. An overview of those models is presented in reference [31].

When force curves are continuously recorded and meanwhile the x-y-scanner is moved, a map of force curves, a so-called force map, can be recorded. It provides information about mechanical properties on different spots of the sample, with a well-defined scanning area and resolution. This allows to analyse a cross-section of adsorbed microgels and compare elastic moduli of different areas (i.e., in the middle or at the edges). A force map also provides (low resolution) height information derived from the point of contact in the force curves.

### 2.2. Dynamic Force Measurements

The complex dynamic modulus $G^{*}=G^{\prime }+\;iG^{\prime \prime }$ can be obtained from dynamic force measurements. In contrast to static force measurements, the tip does not immediately retract after a certain force has been applied, but oscillates in the sample. The ratio of this oscillatory stress to the detected strain gives the dynamic modulus. This method has been first used in biophysics, where it was applied to cells [36,37,38]. After studies on both non-cross-linked and cross-linked polyelectrolyte multilayers [39] it has also been used for measurements on microgels [29,30].

The complex modulus consists of a real part, the storage modulus ($G^{\prime }$) and an imaginary part, the loss modulus ($G^{\prime \prime }$). Dynamic force measurements allow to separately obtain both parts. $G^{\prime }$ represents the elastic properties and $G^{\prime \prime }$ represents the viscous properties of the sample. A sample which acts perfectly elastic would give zero phase shift between force and indentation, thus giving only a real part for the complex modulus. On the other hand, a purely viscous sample would give a phase shift of 90°, leaving only an imaginary part for the complex modulus. Viscoelastic samples give a phase shift between 0° and 90° according to the respective influence of elasticity and viscosity. The quotient of $G^{\prime \prime }$ and $G^{\prime }$ is called loss tangent and gives information about whether elastic or viscous behaviour is dominating. It is the tangent of the phase shift.

**Figure 1:**
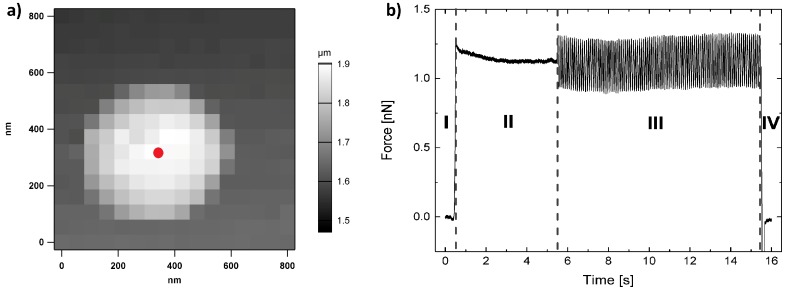
(**a**) Height profile from force map on microgel particle. The red dot indicates the location of the tip, where dynamic force measurements are performed; (**b**) Example force against time plot during a dynamic force measurement. Taken from [30].

For dynamic force measurements on microgels, the gels first have to be located on the substrate. Therefore, the sample is scanned, and then a standard force map is recorded in the area around a microgel particle. From the height profile, the exact centre of the particle can be identified, which is where the tip is then placed for dynamic measurements as shown in Figure 1a. As shown in Figure 1b, the tip is extended to the surface until a certain force is exerted on the cantilever (area I in Figure 1b). This area can be used to calculate *E*, just as in static measurements. Then, the tip is left to equilibrate for some time (area II), where it sinks a little further into the gel. Afterwards, the cantilever starts with a sinusoidal oscillation with a frequency well below the resonance frequency of the cantilever (area III). Finally, the cantilever is retracted from the microgel (area IV). Force and indentation signals are used for data analysis.

The Hertzian contact mechanics model [24,40] allows the calculation of the complex modulus of the sample by

(4) $$G^{*}=G^{\prime }\left(\omega \right)+iG^{\prime \prime }\left(\omega \right)=\frac{1-\nu }{3\delta \tan(\phi )}\frac{F(\omega )}{\delta (\omega )}$$

with the Poisson ratio ν (0.5), the indentation depth δ, and the half tip cone angle ϕ. F(ω) and δ(ω) are the force and indentation signals recorded during the oscillation of the cantilever, which are given by

(5) $$\frac{F(\omega )}{\delta (\omega )}=\frac{A_{F}\left(\omega \right)}{A_{\delta }\left(\omega \right)}e^{i(\varphi_{F}\left(\omega \right)-\varphi_{\delta }\left(\omega \right))}$$

with the respective amplitudes A and phases φ of the force and indentation signals which are fitted with a sine function. The shape of the indenter is assumed to be conical here because of the larger indentation depth compared to static force measurements. Larger indentations are necessary for dynamic force measurements because the oscillation amplitude should be small compared to the indentation.

## 3. Nanomechanics and Nanorheology: Effect of Different Parameters

Many parameters affect the mechanical and rheological properties of adsorbed microgels. In the following they are divided into outer (or external) parameters (Section 3.1) and inner parameters which are related to the parameters which are fixed during synthesis (Section 3.2). In addition, the microgels can be tailored by incorporation of (inorganic) metal nanoparticles. The hybrid microgels can interact then with external fields which is described in Section 3.5.

### 3.1. Outer Parameter

Here, mainly changes in temperature (Section 3.1.1) and solvent compositions (cononsolvency effect, Section 3.1.2) are described. Another important outer parameter is the pH. Since a pH change leads to changes in the electrostatics, it will be discussed together with the charge density below in Section 3.3.

#### 3.1.1. Effect of Temperature

**Figure 2:**
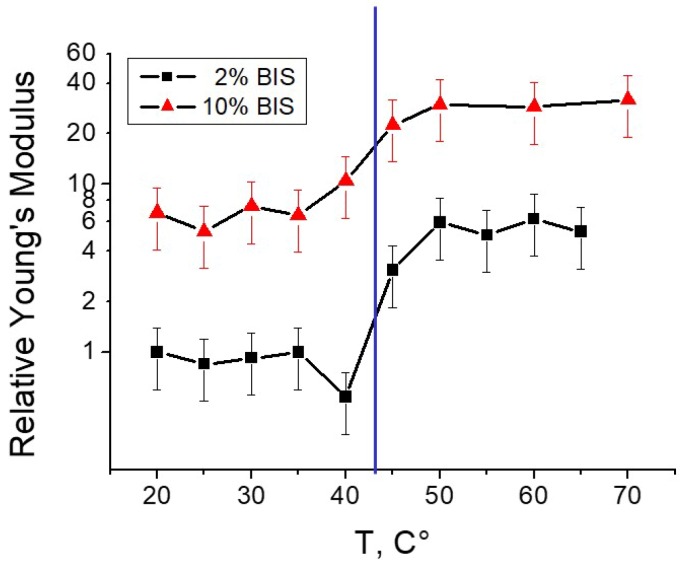
PNIPAM microgels copolymerised with about 2.5% AAc. The amount of cross-linker is either 2% or 10%. Taken from [21].

For PNIPAM microgels, the most prominent parameter for volume changes is the temperature as mentioned in the introduction. Around the VPTT, the microgels shrink which leads to densification of the polymer matrix and to a higher elastic modulus measured by AFM indentation. Figure 2 shows an example of PNIPAM gels cross-linked by BIS.

In this example the increase in elastic modulus with increasing temperature is in the order of factor 5–10. Below in Section 4.2 it will be shown that the increase in elastic modulus caused by temperature increase can be tuned by choosing different amounts and types of crosslinkers. Furthermore, it is shown that the point of inflection of the E-modulus curve is at a temperature above 40 °C which is well above the VPTT. This phenomenon will be addressed below as well as the effect of cross-linker (see e.g., Section 3.2). Some examples for applications of temperature sensitive changes of the elastic modulus are given in Section 4.

#### 3.1.2. Effect of Solvent Composition: The Cononsolvency Effect

Besides the well-studied temperature effect, there are other stimuli that can influence the swelling degree of PNIPAM microgels. One phenomenon is the cononsolvency effect, which occurs in ternary mixtures of PNIPAM with water and organic solvents like short-chained alcohols (methanol, ethanol, or (iso-) propanol) [41,42,43,44]. Both water and alcohol are good solvents for PNIPAM, but adding a small amount of alcohol to an aqueous solution of PNIPAM microgels leads to collapse [29,30,45]. Further addition of alcohol causes a reswelling. The VPTT of microgels decreases with the addition of small alcohol fractions [29,30,42,43,46,47].

**Figure 3:**
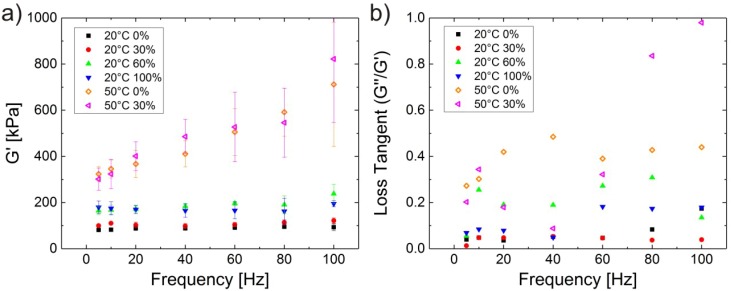
(**a**) Storage modulus $G^{\prime }$ and (**b**) loss tangent $G^{\prime \prime }/G^{\prime }$ of P(NIPAM-*co*-AAc) microgels for various ethanol concentrations at 20 and 50 °C at an indentation force of 2 nN. The microgels were spin-coated from aqueous solutions onto gold surfaces. Taken from [30].

Dynamic force measurements have been carried out with AFM on P(NIPAM-*co*-AAc) microgels adsorbed at gold surfaces in different ethanol concentration below (20 °C) and above (50 °C) the VPTT [30]. Microgels with charged AAc comonomers have been used because electrostatic repulsion increases the size of those microgels compared to pure NIPAM. This reduces the influence of the surface on the results. The microgel volume at 20 °C is minimal between 30 and 50 vol % ethanol. Figure 3 shows the storage modulus $G^{\prime }$ and the loss tangent $G^{\prime \prime }/G^{\prime }$ for varying temperatures and ethanol concentrations. It is known from temperature dependent measurements in water that the elastic modulus of PNIPAM microgels increases when particles shrink with increasing temperature [16,21]. Accordingly, the highest storage modulus $G^{\prime }$ is observed at 50 °C, both in 0 and 30 vol % ethanol. The breaking of hydrogen bonding between PNIPAM and solvent molecules at *T* > VPTT leads to a smaller number of solvent molecules remaining within the collapsed structure. Therefore, more monomer-monomer contacts occur, and the gel becomes stiffer. The solvent composition has no significant influence on the complex modulus at 50 °C, so the cononsolvency effect plays no role at such a high temperature.

Theory predicts a more pronounced frequency dependence of $G^{\prime }$ for materials with a higher loss tangent [48]. This fits to the high loss tangent at 50 °C, as PNIPAM chains are rubbing against each other in the absence of solvent molecules. Thereby they are causing a higher friction and a higher loss modulus $G^{\prime \prime }$. The loss tangent for the microgels at 20 °C is lower, with a maximum at 0.3, which implies a behaviour dominated by elasticity rather than viscosity.

Regarding the cononsolvency effect, $G^{\prime }$ of the microgels at 30 vol % ethanol, where their volume is minimal at 20 °C, is not significantly decreased compared to $G^{\prime }$ in pure water. This finding supports the theory that preferential adsorption of alcohol molecules at PNIPAM in comparison to water is the cause for cononsolvency [49,50,51,52,53]. The gel shrinks because of the formation of loops held together by ethanol bridges. There remains however a rather large number of solvent molecules, which efficiently lubricate the polymer network. The microgel subsequently remains softer in the low-temperature cononsolvency case than in the high-temperature case.

At 20 °C the highest moduli were surprisingly observed for 60 and 100 vol % ethanol, i.e., the systems with the highest ethanol content, whereas it was lower for 30 vol % despite the smaller size. This leads to the assumption that the water which is present in the microgel is mainly responsible for its softness, so it might act like a plasticiser for PNIPAM. There is no direct correlation between size (or swelling degree) and elastic modulus or shear modulus, but they also depend on the microscopic interaction between solvent and gel. The larger binding energy of PNIPAM to ethanol compared to water might play a role here.

From the force curves obtained during the indentation process of the dynamic AFM measurements, the elastic modulus *E* could be extracted as well. For homogeneous and isotropic materials, *E* is connected to $G^{\prime }$ by the relation E=2G(1+ν)=3G for a Poisson ratio ν of 0.5 [54]. The obtained $E/G^{\prime }$ values are between 1.27 and 1.57, which is lower than expected. This is indicative of the finite size and the inhomogeneous structure of the microgels, which consist of a highly cross-linked core and a weakly cross-linked shell [55,56].

### 3.2. Composition of the Microgels (Inner Parameters)

This section starts with a description of the influence of the amount of cross-linker (Section 3.2) and its distribution within the microgel (Section 3.3). Furthermore, the effect of electrostatic repulsion within the microgel is considered, either caused by the copolymerisation with charged monomers during synthesis or/and the post-treatment with different pH.

#### Effect of Cross-Linker Content

Figure 2 shows the effect of the amount of cross-linker (BIS) on the mechanical properties of a PNIPAM microgels. In this example, the BIS content was 2% and 10%, respectively. As expected, the microgels are stiffer with increasing BIS content. The temperature effect is a bit less pronounced for the microgel with the higher cross-linker content. These results correlate well with a lower swelling ability for microgels with a higher amount of cross-linker which was found by many groups for microgels both in the bulk phase and at the surface.

**Figure 4:**
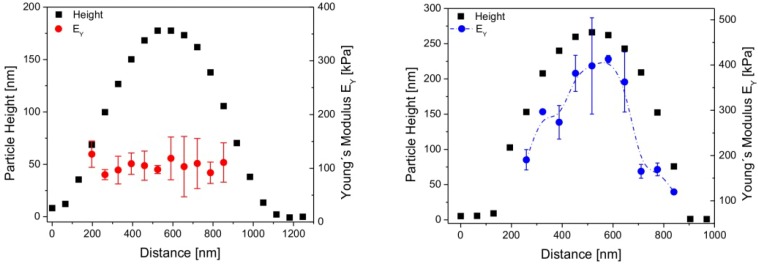
Particle heights and the corresponding Young’s moduli versus the particle cross section of cross-linker-free microgels (**left**) and microgels with 5 mol% cross-linker (**right**). Taken from [28].

In addition, as already mentioned above, the transition temperature of the elastic modulus (point of inflection) is shifted to higher temperatures with respect to the VPTT. The elastic modulus transition temperature will be abbreviated by EMTT in the following. A first explanation for the mismatch between VPTT and EMTT was that this phenomenon is related to a kind of core-shell structure of microgels if they are synthesised by the batch method, i.e., if cross-linker and monomers are put altogether at the same time into the reactor. Due to a faster reaction kinetics of the cross-linker in comparison to the monomer at the beginning of the reaction, more cross-linker is consumed at the beginning of the reaction than later on. This results in a denser core and a more fluffy shell. This internal core-shell structure was described by several groups for different microgels [57,58,59]. It was also proved by force maps which gives the elastic modulus over the cross-section [16,21,28] as shown in Figure 4. In the following, the microgels synthesised by a one batch method resulting in a denser core and a more fluffy shell are called *core-shell microgels*.

During the AFM indentation experiments (static or dynamic), mainly the outer part of the microgel is analysed (see Section 2). Therefore, if the core and the shell shrink at different temperatures due to their different polymer density this might be an explanation for the mismatch between the VPTT and the EMTT. So far, there is no clear proof that differences in the density could result in such a large difference in VPTT. Papers addressing the effect of PNIPAM concentration on the LCST could find only an effect of a few Kelvin on the shift in VPTT [60]. However, if the density gradient has a certain effect on the shift between VPTT and EMTT, it should be vanished or at least reduced for microgels with a homogeneous density. This issue will be addressed more in detail in the following.

*Cross-linker-free microgels:* One possibility to reduce the radial density gradient within microgels is to synthesise self-crosslinked PNIPAM microgels without addition of a cross-linker [61,62,63]. This results in ultra-low cross-linked (≈0.25 mol%) microgels. Apart from PNIPAM, cross-linker-free oligo(ethylene glycol)-side-chain microgels (PEG) have been synthesised, and their mechanical properties have been analysed by AFM force measurements [28]. PEG is appreciated for a low toxicity, good biocompatibility and anti-fouling behaviour.

The elastic modulus at the centre of the cross-linker free microgels was five times lower than for gels with 5 mol% poly(ethylene glycol diacrylate) (PEGDA) which acts as cross-linker. This explains the enhanced spreading of cross-linker-free microgels. Force maps have been recorded on both types of microgels, as shown in Figure 4.

Microgels with cross-linker show the well-known maximum in elastic modulus at the particle centre, which is by a factor of 2.2 larger than the modulus at the rim. Microgels without cross-linker show similar values throughout the whole particle. Therefore, cross-linker-free microgels are assumed to have a homogeneous inner structure.

### 3.3. Homogeneous It vs. Core-Shell Microgels

**Figure 5:**
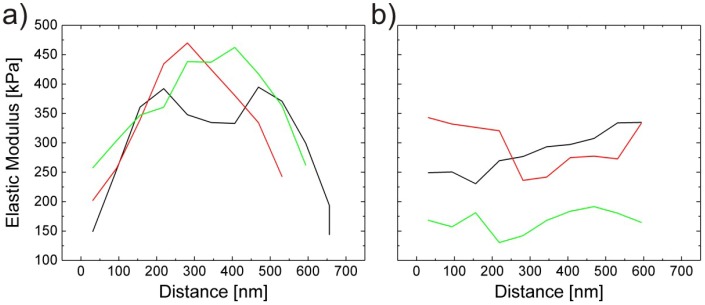
Elastic moduli of (**a**) core-shell microgels (batch method) and (**b**) homogeneous microgels (feeding method) measured across the cross section (force map) for three individual microgel particles. Taken from [65].

Another strategy to synthesise microgels with a homogeneous polymer density across the microgel is to compensate the faster reaction kinetics of the cross-linker by feeding monomers into the reactor during the reaction [64] (feeding method). The overall composition of the two microgel species was the same with 2.5% BIS and 2.5% of the cross-linker allylamine. Figure 5 shows force maps on core-shell microgels synthesised by the batch method and on microgels synthesised by the feeding method.

The elastic moduli of core-shell particles show a clear maximum at the centre of the particle, and the elastic moduli decrease as the AFM tip approaches the microgel shell as described above. However, for microgels synthesised by the feeding method, there is no significant change of elastic moduli across all three tested particles. Therefore, these microgels are assumed to have a homogeneous density distribution. Average E-modulus values are however roughly in the same order for core-shell and homogeneous microgels.

**Figure 6:**
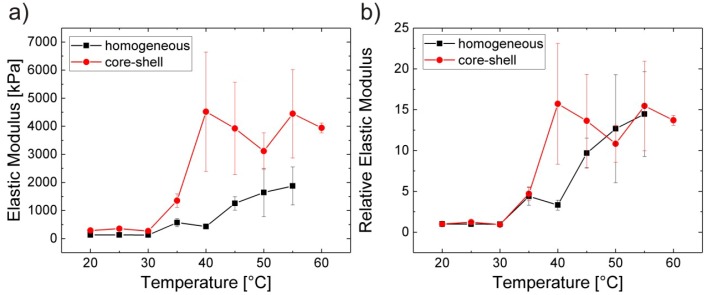
Elastic moduli of core-shell and homogeneous microgels depending on temperature: (**a**) absolute values and (**b**) normalised with respect to the elastic modulus at 20 °C. Taken from [65].

Increase in temperature leads to a stiffening of the microgels as shown in Figure 6.

The values for all particles have been obtained at the centre of the particle. The core values are more than twice as high as the values of homogeneous gels below the transition temperature. Above the transition temperature, the difference between both types of microgels is even more pronounced. The E-modulus of the core of the core-shell microgels increases by a factor of about 10, while the E-modulus of the homogeneous gel increases by a factor of about 6. For easier comparison the values have been normalised with respect to the elastic modulus of the respective gel at 20 °C. The trend looks very similar for both microgels: The transition zone expands a temperature regime from about 30 °C up to 45/50 °C. This is a clear hint that the VPTT is lower than the EMTT or both types of microgels, i.e., core-shell and homogeneous microgels. This leads to the conclusion that the difference between VPTT and EMTT is not related to the density distribution across the microgel.

It might be referable to microscopic differences in cross-linker density, with some randomly distributed small domains with low polymer density retaining a certain softness. On the other hand, the breaking of hydrogen bonds between water and polymer during the shrinking process is followed by the formation of intra- and inter-chain hydrogen bonds between PNIPAM monomers. This is supposed to strengthen the network, and would increase *E* and $G^{\prime }$. The formation of those bonds might not be completed until around 50 °C, leading to the observed continuous increase of elastic and storage moduli up to this temperature.

Additionally, rheological temperature sweep measurements have also been done on PNIPAM macrogels, where $G^{\prime }$ increases up to 50 °C [66]. The transition temperature lies above the VPTT for core-shell and homogeneous microgels, as well as for macrogels. These results on macrogels also support the assumption that the cross-linker distribution is not responsible for the difference between VPTT and EMTT.

Referring to the mismatch between the degree of swelling and the rheological properties in different solvents, the same dynamic measurements were done for homogeneous microgels as in Section 3.1.2. Again, the smallest microgel appears at 30 vol % ethanol, and it does not give the highest storage modulus $G^{\prime }$. The highest value for $G^{\prime }$ is determined again for 60 vol % ethanol. Overall, the results show once more that there is no direct relationship between particle size and stiffness, but that the stiffness is very much influenced by the micro-structure.

The loss tangent shows the same trend as for core-shell microgels: The higher the storage modulus of the system, the higher is the loss tangent, which means that the viscous influence is higher for stiffer particles. Values for the loss tangent are higher for homogeneous microgels compared to core-shell microgels roughly by a factor of 2. Obviously, the viscous contribution is higher for homogeneous microgels than for core-shell particles. This might be caused by the larger size of the homogeneous microgels in comparison to their core-shell counterparts of the same composition.

#### Effect of Charge Density

**Figure 7:**
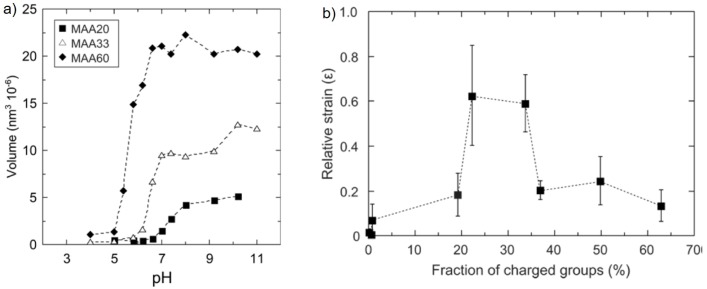
Microgels copolymerised with different amounts of methacrylic acid (MAA): (**a**) Volume of microgels in bulk solution and (**b**) microgel deformability, expressed as relative strain, as a function of the fraction of charged groups within the microgels. Taken from [15].

Electrostatic swelling transitions of methacrylic acid (MAA)-based microgels (≈100 nm diameter) covalently bound to silica surfaces were investigated as a function of pH and microgel charge density [15]. They swell monotonously with increasing charge density, either by increasing the MAA content of the microgel or by increasing pH as shown in Figure 7a.

In contrast to the swelling behaviour, the mechanical properties show a non-monotonous change with increasing charge density. The swelling leads to a softening of the microgels up to intermediate charge densities (20%–35% charged MAA monomers). They are deformed up to around 60% of their original height (relative strain of ≈0.6) when an 800 pN force was applied. The elastic modulus shows its minimum at intermediate charge densities. A further increase in charge density leads to an increase in the elastic modulus due to the electrostatic repulsion which becomes sufficiently high to overcome the applied load from the cantilever tip (see Figure 7b). Consequently, the deformability of the microgel decreases again. This is an example of the complex relation between swelling and elastic behaviour. It has further been shown that a low applied force of 2 nN only pushes the polymer chains elastically. In contrast, a higher force of 5 nN leads to energy dissipation, which means that water is pushed out or redistributed within the gel. These studies show that there are two counteracting effects induced by increasing internal electrostatic repulsion acting on the microgel stiffness: the decrease in stiffness due to swelling with water and the direct repulsion between the charges which counteracts the external force.

In addition, PNIPAM microgels are larger when NIPAM is co-polymerised during synthesis with charged comonomers like acrylic acid (AAc) [16] or allylacetic acid (AAA) [67]. Due to the higher water content the elastic modulus decreases with increasing amount of comonomer, which might be the same effect as for MAA–containing microgels up to an intermediate charge density. As expected, the elastic modulus increases with an increase in temperature. Again the VPTT is lower than the EMTT, but the difference increases with increasing charge density. This might be a hint that the difference between the VPTT and the EMTT is correlated with charge effects.

**Figure 8:**
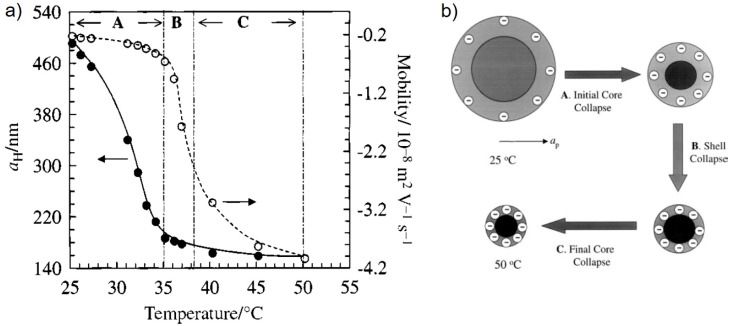
(**a**) Electrophoretic mobility (open circles) and hydrodynamic radius (closed circles…) as a function of temperature for PNIPAM microgel dispersions in aqueous $10^{3}$ M NaCl. The labels A, B and C refer to different stages of particle collapse; (**b**) Proposed model for the temperature-dependent de-swelling of PNIPAM microgel particles. The particle is considered to have a lightly cross-linked shell that contains most of the charged groups present. The swollen particle radius is a core collapse occurring in the first stage (A) followed by partial shell collapse at higher temperature (B). Further collapse of the core occurs at higher temperatures (C), leading to a hard particle surrounded by a non-rigid, partially collapsed, shell at ca. 50 °C. Taken from [57].

This hypothesis is strongly supported by ζ-potential measurements which give information about the change of charge density of the microgel with temperature variation. Several studies have shown that the charge density increases with increasing temperature and shrinking of the PNIPAM microgel (e.g., [68]). It is explained by reduction in surface area of the microgel and by a rearrangement of the gel structure which expresses the charges more towards the microgel surface. Figure 8 shows the hydrodynamic radius and the electrokinetic mobility of PNIPAM microgels in the bulk solution as a function of temperature.

In this example the negative charge is caused by the carboxy groups from the initiator residues in the microgel. The electrokinetic transition temperature (ETT) is also shifted towards higher temperature with respect to the VPTT, as described for the EMTT above. This might be a hint for a strong correlation between rearrangement effects and the change in mechanical properties and which takes place at temperatures above the VPTT. Figure 8b shows the proposed model for the assumed rearrangement. Obviously, such a rearrangement occurs even in homogeneous microgels.

*Amphiphilic comonomers:* While copolymerisation with AAc gives a monotonous decrease in the elastic modulus, copolymerisation with charged monomer containing a short aliphatic chain leads to a non-monotonous change in mechanical properties of the microgels. As an example, PNIPAM microgels copolymerised with different amounts of allyl acetic acid (AAA) is given [67]. At low amount of AAA, the elastic modulus decreases as for AAc. With further increase of comonomer content, the elastic modulus increases for AAA while it further decreases in the case of AAc. Obviously, two counteracting effects occur for the AAA. At low amount, the charge effect dominates and at higher amount of AAA the influence of the aliphatic chain becomes pronounced. It is assumed that the aliphatic chains act as additional cross-linkers which stiffen the microgel.

### 3.4. Composite Microgels

Two different types of composite microgels are considered. The first section addresses fully organic core-shell microgels. In contrast to the core-shell microgels described above, core and shell are chemically different, now. The second section focuses on microgels which are doped with metal nanoparticles.

### 3.5. Microgels with a Stimuli-Insensitive Shell

Here, core-shell particles are considered with a temperature-sensitive PNIPAM core and a temperature-insensitive polyacrylamide (PAAM) shell [27]. In other words, the PAAM shell acts a bit like a “corset” which was explained earlier by Richtering and coworkers [69].

**Figure 9:**
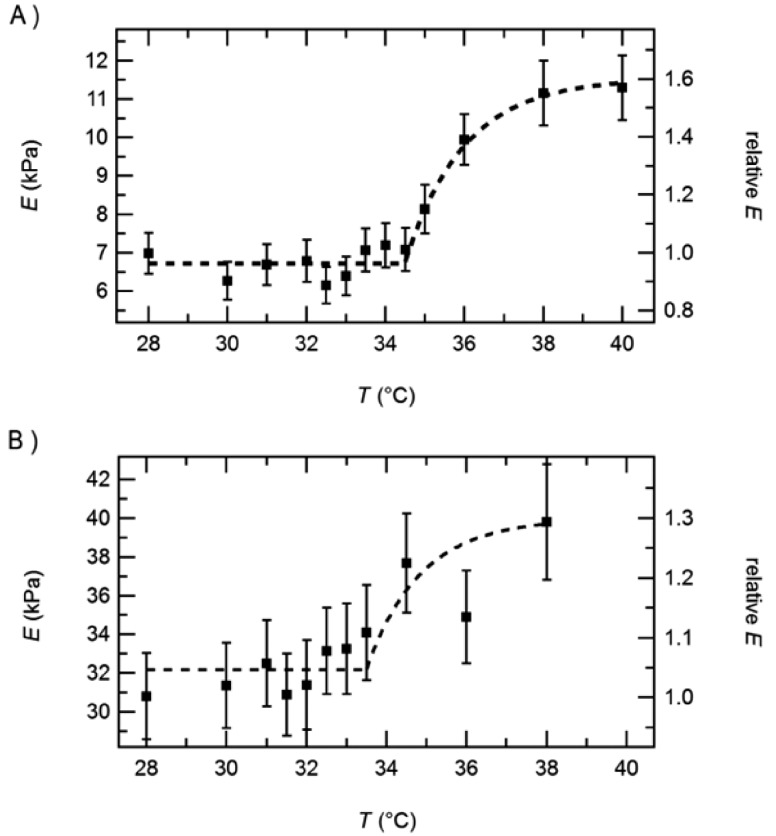
Temperature-dependent elastic moduli of PNIPAM-PAAM core-shell particles with thin (**A**) and thick (**B**) PAAM shell. Taken from [27].

Figure 9 shows the E-modulus of gel particles which were produced by a microfluid device. Therefore, they are much larger (with radii up to 40 μm) than the ones described so far in the review. The larger size allowed indentation measurements with a colloidal probe (radius of 2 or 23 μm) attached to the cantilever which gives more averaged information about mechanical properties than with a tip as an indenter [25]. The gel particles were produced with two different shell thicknesses. Up to the VPTT, the E-modulus of both microgels is temperature-independent. Upon temperature increase above the VPTT, the thermoresponsive PNIPAM core collapses, which leads to an increase in the E-modulus for both types of particles, as shown in Figure 9.

The changes in E-modulus as well as in size are more pronounced for the microgels with a thinner shell. It is assumed that for thin shells, the shell volume decreases above the VPTT because the shrinking core is able to drag the chains of shell into the core. This increases the polymer concentration and subsequently the elastic modulus. For thicker shells, the elastic modulus of the gel particle is already higher below the VPTT. With increasing temperature the overall particle volume is hardly reduced. The mutual effects between both polymers at their common interface are important for the temperature response of the whole particle.

**Figure 10:**
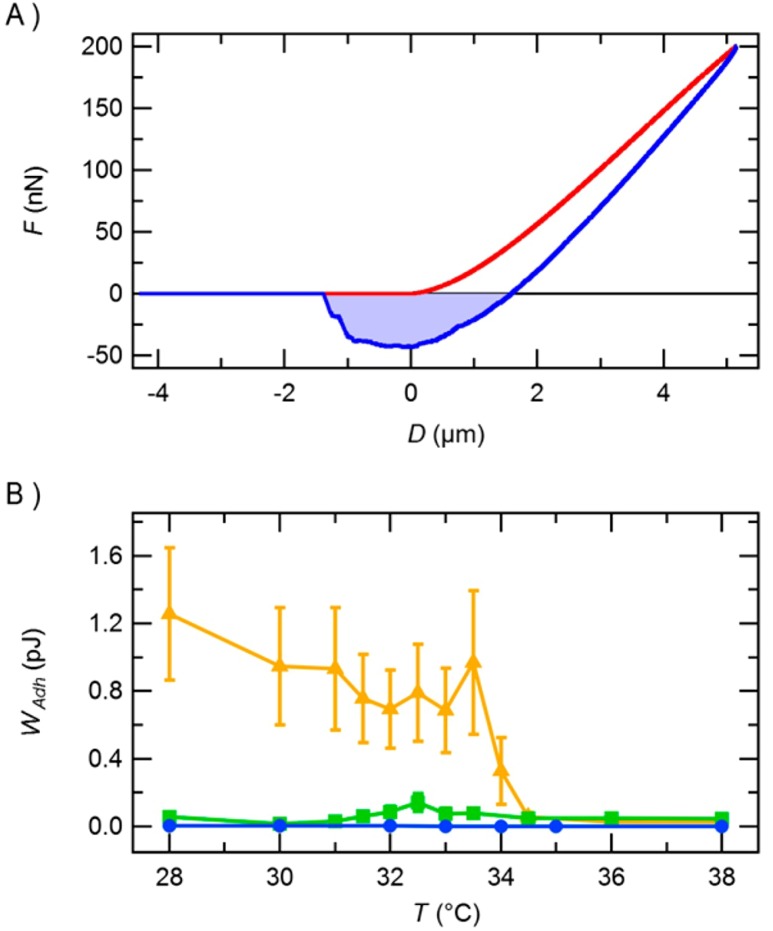
(**A**) Force curve during approach (red) and retraction (blue). The light blue area represents the work of adhesion; (**B**) Temperature-dependent work of adhesion for plain PAAM (blue circles), plain PNIPAM (orange triangles), and PNIPAM-PAAM core-shell (green squares) microgels. Taken from [27].

Such gel particles are interesting for applications where a reduced adhesion is desired. The work of adhesion is the total energy needed to pull the probe off the surface, experimentally accessible as the area enclosed by the negative part of the force curve and the abscissa, shown in Figure 10A.

Plain PNIPAM microgels show a high work of adhesion below the VPTT, and a decreased adhesion above the VPTT. In contrast, the work of adhesion for PNIPAM-PAAM core-shell microgels, as well as plain PAAM microgels, is low in the whole temperature range, as shown in Figure 10. This means that the change in elasticity has been successfully decoupled from a change in the microgel surface properties (here: adhesion).

#### Microgels Doped with Metal Nanoparticles

The outer parameters mentioned above act quite slowly due to changes of liquids or heating up/cooling down of the liquid. Therefore, external fields like light, magnetic or electric field are more suitable triggers. In order to obtain microgel particles which can be triggered by external fields, one option is to embed metal nanoparticles within the microgels. For instance, gold nanoparticles (AuNP) interact with light and can be embedded into microgels [70,71,72,73,74]. The AuNP can act in two directions: (1) Changing the optical properties of the microgels and (2) serving as hotspots which can let shrink locally the PNIPAM matrix. In addition, magnetic nanoparticles (MNP) can be added to microgels [26,75,76,77,78,79,80,81,82,83,84,85,86,87,88,89].

On one hand the mechanical properties of the microgel affect the uptake of nanoparticles, on the other hand the loading affects the mechanical properties of the microgels.

It has been shown for macroscopic gels loaded with MNP (ferrogels) that the elastic modulus increases in the presence of an external magnetic field [90,91]. This is caused by regions in the polymer which are compressed by MNP, which tend to form chains in a magnetic field. Theoretical studies on magneto-sensitive elastomers predict an increase of the shear modulus *G* irrespective of the MNP distribution. In contrast, an increase of the elastic modulus is only predicted for plane-like MNP arrangements, whereas E decreases for chain-like or isotropic arrangements of MNP [92]. However, the assumed perfect rectangular MNP arrangement and the lack of flexibility in these considerations might cause deviations from experimental observations. Shape-anisotropic unbound magnetic particles can be used in microrheological measurements to probe the shear stiffness in hydrogels locally [93,94]. The results of such measurements reflect the macroscopic properties if the size of the probe particle is large compared to the molecular microstructure [95].

**Figure 11:**
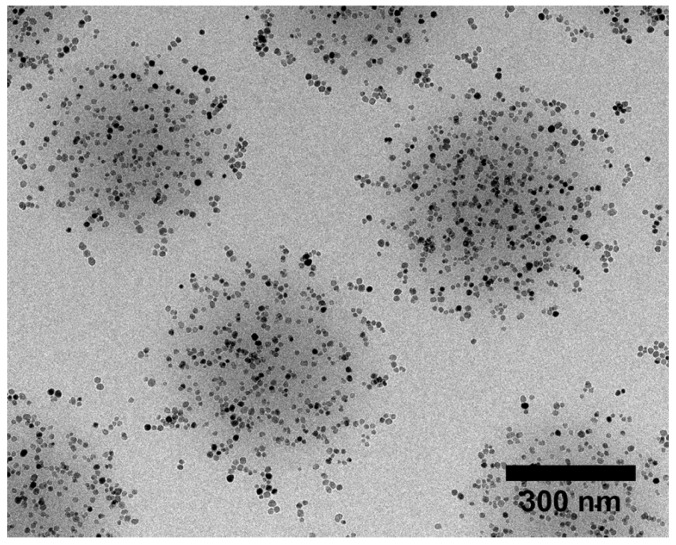
TEM image of P(NIPAM-co-acrylic acid) microgels with CoFe_2_O_4_ MNP (coated with polyacrylic acid).

Microgels loaded with MNP, which will be called magnetic microgels in the following, have been subject to static AFM force measurements [26,89]. A transmission electron microscopy (TEM) image of P(NIPAM-*co*-acrylic acid) microgels with CoFe_2_O_4_ MNP (coated with polyacrylic acid for stabilisation) is shown in Figure 11 to illustrate the loading.

So far two conclusions can be drawn from indentation measurements: First, the pure microgel has a higher elastic modulus than the magnetic microgel. This effect might be related to the increase in the microgel size after loading with MNP. This increase is probably caused by repulsion between the MNP, which let the meshes inside the microgel grow. Another or additional reason might be that the charged MNP increases the osmotic pressure within the microgel. Hence, more water enters the gel. The same effect was also observed for charged gold nanoparticles. Second, there is no measurable influence of an external magnetic field on the elastic modulus of the magnetic microgels. Obviously, spherical MNP are not the optimum and bound anisotropic MNP might deform the gel in a stronger manner, which might result in a switchable elastic modulus.

## 4. Applications

Finally a few application-oriented examples are given, where mechanical properties of microgels play a role.

### 4.1. Microgels Acting as Cellular Micro-Environments

Cells are affected by the stiffness of the extracellular matrix (ECM) which surrounds them. To study the role of this stiffness in situ, cells have been embedded in large microgels (*r* = 20 μm) with adjustable mechanical properties [96]. Cell-loaded microgels have been fabricated by microfluidic emulsification of agarose solutions with carcinoma associated fibroblast (CAF) cells and subsequent gelation of the precursor droplets by thermosetting. The Young’s modulus was measured by AFM force measurements with a tipless cantilever, which was applicable because of the large size of the microgels. Using tipless cantilevers is less invasive, and the results agreed well with compression test measurements on macroscopic gels.

The Young’s modulus at room temperature increased from 0.62 ± 0.05 to 20.2 ± 0.5 kPa by increasing the agarose concentration during synthesis from 1.0 to 5.0 wt %. The stress-relaxation time, which has been obtained from a dwell measurement of the cantilever on the microgel, decreased from 471 ± 41 to 106 ± 18 ms in the same concentration range. The decrease occurred due to the combined effect of the relaxation of dangling ends of agarose, the dynamics of moderately cross-linked network fragments that had length distribution, and the topological constraint release effects of network strands.

Under physiological conditions, the Young’s modulus of the agarose microgels was significantly decreased, e.g., from 20.2 ± 0.5 kPa to 4.6 ± 0.3 kPa for 5.0 wt %. This was ascribed to structural changes in the gel network [97]. The Young’s modulus of the microgels was not affected by loading with cells, neither by loading with rigid poly(methyl methacrylate) (PMMA) analysed as a reference system. The lack of influence of the loading with cells has been explained by a collagen shell, which is expressed by the cells. Collagen has a Young’s modulus which is significantly higher than that of agarose [98].

**Figure 12:**
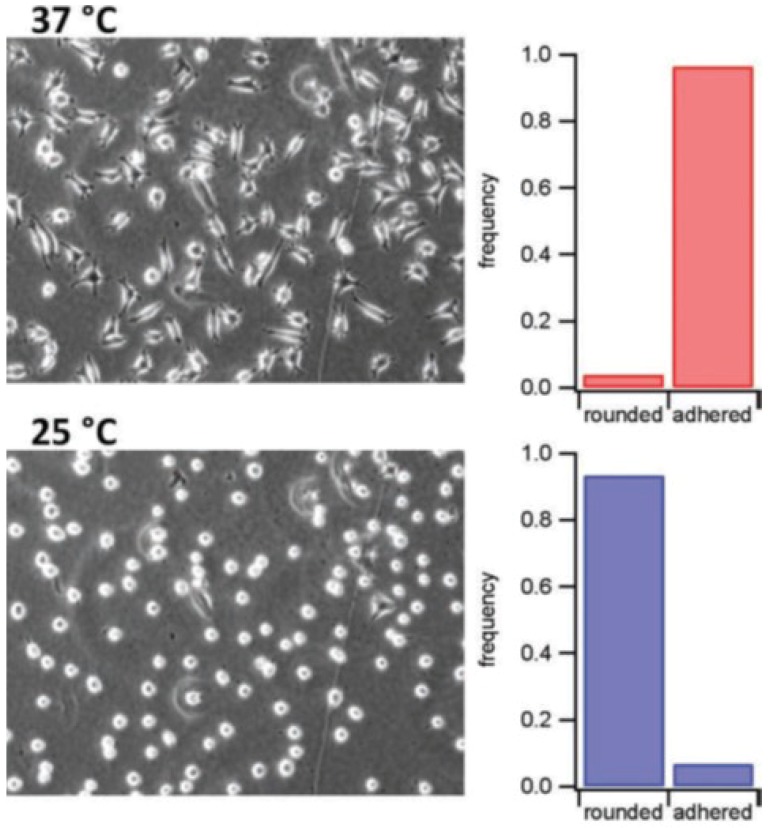
Phase contrast microscopy images of cells 48 h after incubation at 37 °C (95% of the cells adhere) and 20 min after cooling the surface to 25 °C (95% of the cells detach). Taken from [99].

Another direction of applications is towards switchable mechanical properties to control the cell adhesion. Cells prefer the adsorption on ‘‘hard’’ material. Of course from anti-fouling studies it is known that it is not that simple. Just making the surface soft is not enough to prevent adsorption of cells and proteins. Nevertheless, a proof of concept shows that switching the elastic properties of a PNIPAM coated surface just by varying the temperature is very efficient for the control of cell adhesion. Figure 12 shows that below the VPTT, when the PNIPAM microgels are soft the cells prefer a small contact area with the substrate and they can be easily rinsed away. Above the VPTT, the cells adhere stronger at the surface and form a larger contact area with the PNIPAM coating.

### 4.2. Tactile Feed-Back Devices

**Figure 13:**
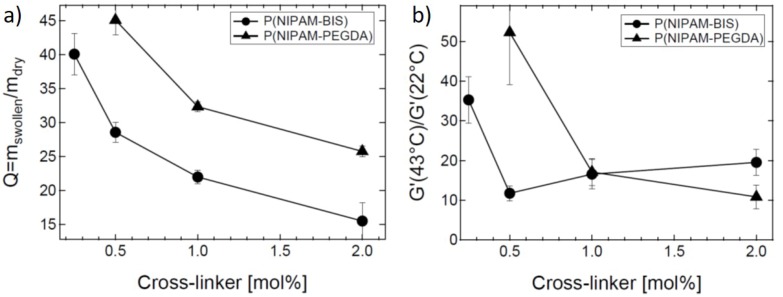
Comparison of PNIPAM microgels cross-linked with BIS (circles) or PEGDA (triangles) with respect to their (**a**) swelling behaviour and (**b**) ratio of the storage moduli $G^{\prime }$ at 43 °C and 22 °C. Data taken from [66].

Soft and smart surfaces are omnipresent for operating everyday touch-controlled devices like mobile phones or tablets. For visually handicapped persons or situations in daily life where a direct eye contact to the screen is not possible, a programmable tactile surface would be helpful. For this purpose, thin stimuli-responsive hydrogel films which show a programmable reversible change in elasticity comes into play [100]. The stimuli-sensitive hydrogel layer can be locally stiffened to form tactile buttons resulting in a haptic feedback for the user [101,102]. An example is *GelTouch* [101], a PNIPAM hydrogel, which is transparent and soft when not activated. *GelTouch* can be selectively activated by local heating creating tactile feedback. The aim is to get a strong reversible change in elastic modulus by heating and cooling. Therefore, the gel network architecture has to be tailored. One example is the replacement of the cross-linker BIS by a longer and more flexible one, PEGDA [66,103]. The higher flexibility of the cross-linker leads to higher swelling ability and a higher ratio between the storage moduli above and below the VPTT. For PEGDA-cross-linked microgels, storage modulus at 43 °C can be more than 50 times higher than for 22 °C as shown in Figure 13.

### 4.3. From Pickering Emulsions to Mickering Emulsions

**Figure 14:**
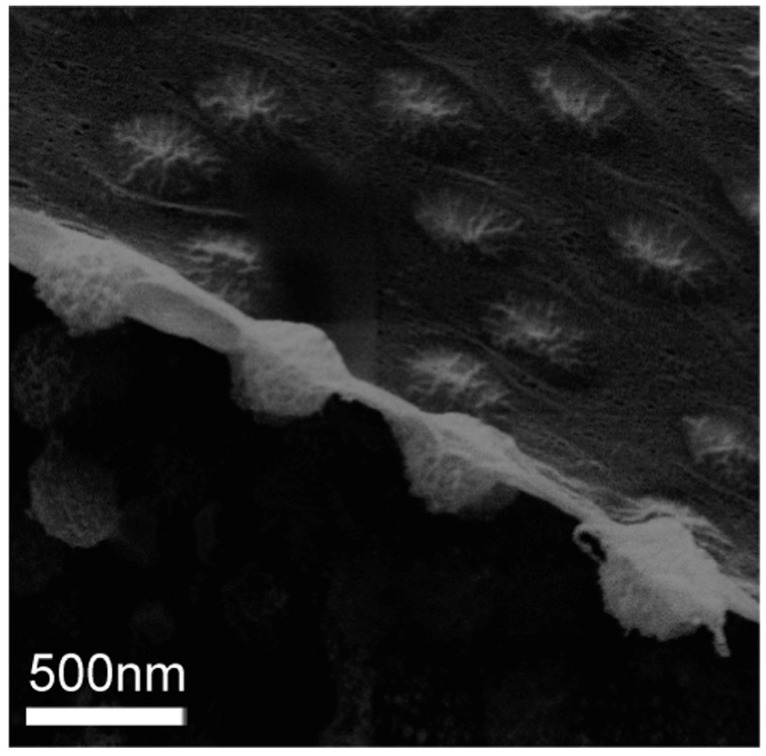
PNIPAM particles at an oil–water interface, imaged using cryo-SEM. Taken from [104].

So far, the mechanical properties of adsorbed microgels were addressed at solid interfaces. It was mentioned that the mechanical properties affect the deformation of the microgels at the interface which is mainly monitored in the vertical direction. The direction lateral to the surface is often not that much affected and often dominated by the dimension of the microgel in the bulk phase before adsorption. At a liquid interface, the lateral dimension can drastically change and the microgels can be strongly stretched determined by the surface tensions of the three interfaces (microgel-liquid1, microgel-liquid2, liquid1-liquid2) and the mechanical properties of the microgel itself. The visualisation of the stretching was subject of several studies [104,105] as shown in Figure 14. This in turn affects the packing of the microgels and the 2-dimensional elastic properties of the whole interface [106,107].

Emulsions can be stabilised by surfactants or particles. The latter ones are called Pickering emulsions. Besides solid particles, soft particles get more and more impact. They play an important role in food technology (e.g., proteins) and they can make emulsions switchable [108]. Due to the deformability at the liquid interface, the simple rules of Pickering emulsions with respect to hard particles are not valid anymore. Therefore, they were called “Mickering emulsions” [109]. According to our knowledge, a theoretical understanding of Mickering emulsions is still missing. A respective theory should combine surface energy/tension aspects with mechanical properties of the microgels.

## 5. Summary, Conclusions and Outlook

The review addresses the mechanical properties of microgels adsorbed at interfaces. Since they have often a size below 1 μm, a high spatial resolution of the mechanical measurements is required. For this purpose, static and dynamic AFM indentation measurements (so-called force maps) are suitable. For some studies, there is a quite simple correlation between the swelling behaviour of the microgels and the mechanical properties. For instance, with increasing temperature a LCST-type microgel shrinks and its elastic modulus increases. Or the microgels swell with increasing charge density and their elastic modulus decreases.

In contrast to this simple correlation, it has been shown that the transition temperature of the elastic modulus (EMTT) is often shifted towards higher temperatures in comparison to the VPTT. A detailed investigation showed that the polymer density distribution across the microgel does not seem to be the reason since the difference between EMTT and VPTT is observed for core-shell type microgels, homogeneous microgels and macrogels. On the other hand it was observed that the shift between VPTT and EMTT becomes more pronounced for microgels with a higher charge density. In addition, electrokinetic measurements show that the transition from low to high charge density (ETT) with increasing temperature also takes places above the VPTT. This might be an indication for rearrangements of the gel structure (and charges) above the VPTT without any pronounced effect on volume changes.

In cononsolvent systems, it is still unclear why microgels show a maximum in $G^{\prime }$ in 60 vol % ethanol, and not in 30 vol %, where their volume is minimal. Interaction strength of solvent and gel or solvent compressibility might play a role here, as well as the solvent composition inside the gel. The composition of the adsorbed solvents has been shown to differ from the bulk composition [110,111]. A study systematically simulating the composition in the first solvation shell depending on bulk composition and temperature could help provide an explanation for higher moduli of microgels in 60 vol % ethanol despite their smaller size.

To summarise, the correlation between the swelling behaviour and the mechanical properties of microgels is not as simple as expected.

A further task refers to composite microgels. AFM force curves measured with a tip could not be analysed for microgels which are highly loaded with MNP because of their grainy structure. Better results might be achieved with a colloidal probe glued to the cantilever, measuring the average response of numerous microgels. To do this in a defined and reproducible way, however, a closed-packed monolayer or multilayer of magnetic microgels should be prepared.

The review addresses the mechanical and rheological properties of microgels, i.e., properties on a small length scale. On the other hand, properties on a small length scale are also of interest in heterogeneous macrogels. Most gels have defects or inhomogeneities as already described for microgels. Beside the ones which are difficult to avoid during the synthesis process, inhomogeneities might be induced on purpose to change the macroscopic mechanical properties. The question is how these inhomogeneities affect the mechanical properties and if the macroscopic mechanics can be predicted. This requires a deep understanding and control of the relationship between structure, swelling abilities and mechanical/rheological properties of the gels. This is a challenge for synthesis and characterisation as well as for theoreticians. First of all the gel structure has to be controlled during synthesis and has to be known, so that the theoreticians can model it. It is assumed that e.g., the E-modulus is not simply a linear combination of the moduli of different domains of different polymer densities, since the domains affect each other. The question is what type of cross terms have to be used to describe the mechanical properties realistically. Experimentally, one can measure the mechanical and rheological properties of the different domains more or less separately, e.g., by AFM indentation [112].

Beyond these very fundamental and technical questions, mechanical properties of microgels are interesting for many technical applications and for model systems related to biologically relevant questions. Due to their strong swelling ability, microgels can be considered as a model system for an extracellular matrix of organs [113]. As discussed above, cells can sense the mechanical properties of the environment. This becomes important for healing processes of natural tissues if e.g., artificial fibres or tissues are implanted. It is also known that the mechanical properties of the cell environment affect the chemical response of a cell. In this context, it might be of interest to check the impact of microgels on the chemical expression of the cells. As mentioned above, the mechanical properties of microgels can be easily varied. A challenge will be of course that only biocompatible material should be used, which is of course more limited in terms of variation than the materials mentioned in the present review.

For pharmaceutical or biotechnical applications, the microgels should be mechanically robust. For instance, when they are compressed in tablets or when they are used in plug-flow reactors they have to resist high mechanical stress.

Tactile devices are at the beginning of their development and their applications can be go much beyond touchscreens. For instance, feed-back systems might be an interesting field.

Switchable emulsions are interesting for catalysis. Often during reactions water is one of the products. Unfortunately, a changing water/oil ratio might change the efficiency of the reaction, i.e., reducing the yield. Therefore, it is important to release the water. A suitable external trigger could destroy the emulsion. Of course, after switching off the trigger the emulsion should reform itself. To control this process the understanding of the combination of surface energy aspects and mechanical properties at fluid interfaces of soft particles (as microgels) are essential.

The review shows that there are still many open fundamental questions about nanomechanics and nanorheology of (micro)gels, and it shows that it is a broad field with a lot of impact for future applications.
